# Surgical spacer placement for proton radiotherapy in locally advanced pancreatic body and tail cancers: initial clinical results

**DOI:** 10.1186/s13014-020-01731-z

**Published:** 2021-01-06

**Authors:** Dongha Lee, Shohei Komatsu, Kazuki Terashima, Hirochika Toyama, Yoshiro Matsuo, Daiki Takahashi, Masaki Suga, Naoko Nishimura, Kentaro Tai, Masahiro Kido, Yusuke Demizu, Sunao Tokumaru, Tomoaki Okimoto, Ryohei Sasaki, Takumi Fukumoto

**Affiliations:** 1 Department of Surgery, Division of Hepato-Biliary-Pancreatic Surgery, Kobe University Graduate School of Medicine, 7-5-2, Kusunoki-cho, Chuo-ku, Kobe, Hyogo 650-0017 Japan; 2 Department of Radiology, Hyogo Ion Beam Medical Center, 1-2-1 Kouto, Shingu-cho, Tatsuno, Hyogo 679-5165 Japan; 3 Department of Radiation Physics, Hyogo Ion Beam Medical Center, 1-2-1 Kouto, Shingu-cho, Tatsuno, Hyogo 679-5165 Japan; 4 Department of Radiation Technology, Hyogo Ion Beam Medical Center, 1-2-1 Kouto, Shingu-cho, Tatsuno, Hyogo 679-5165 Japan; 5 Department of Radiation Oncology, Hyogo Ion Beam Medical Center Kobe Proton Center, 1-6-8, Minatojimaminami-machi, chuo-ku, Kobe, Hyogo 650-0047 Japan; 6 Division of Radiation Oncology, Kobe University Graduate School of Medicine, 7-5-2, Kusunoki-cho, Chuo-ku, Kobe, Hyogo 650-0017 Japan

**Keywords:** Surgical spacer, Pancreatic cancer, Proton radiotherapy, Dose volume histogram, Radiation toxicity

## Abstract

**Background:**

Particle radiotherapy has increasingly gained acceptance for locally advanced pancreatic cancers owing to superior tumor conformity and dosimetry compared to conventional photon radiotherapy. However, the close proximity of the pancreas to the stomach and duodenum leads to radiation-induced gastrointestinal toxicities, which hinder the delivery of curative doses to the tumor. To overcome this problem, a surgical spacer was placed between the tumor and gastrointestinal tract, and subsequent proton radiotherapy was performed in this study.

**Methods:**

Data from 9 patients who underwent surgical spacer placement and subsequent proton radiotherapy were analyzed. The safety and feasibility of the spacer placement surgery were evaluated; the impact of the spacer on dosimetry was also assessed using dose volume histogram (DVH) analyses, before and after surgical spacer placement.

**Results:**

Surgical spacer placement and subsequent proton radiotherapy were successfully completed in all cases. Surgical spacer placement significantly improved the dose intensity covering 95%, mean, and minimum doses for the gross tumor volume, and the clinical and planning target volume based on the DVH, while respecting the dose constraints of the gastrointestinal tract. Based on the Common Terminology Criteria for Adverse Events, two patients (22.2%) developed gastrointestinal ulcer (Grade 2) at 1 and 35 months, and one patient (11.1%) developed gastric perforation (Grade 4) at 4 months after proton radiotherapy.

**Conclusions:**

Surgical spacer placement in the locally advanced pancreatic body and tail cancers is relatively safe and technically feasible. Comparing radiation plans, surgical spacer placement seems to improve the dose distribution in the locally advanced pancreatic body and tail cancers, which are close to the gastrointestinal tract.

## Background

Pancreatic cancer is now the fourth leading cause of cancer-related deaths in the United States and is expected to become the second before 2030 [1]. Although surgical resection of localized tumors is curative, the majority of affected patients are diagnosed with unresectable locally advanced or metastatic disease [2, 3]. The prognosis of unresectable pancreatic cancer is therefore dismal, and effective treatments are required to improve survival. Patients with unresectable pancreatic cancer can be categorized into two broad groups, namely, those with distant metastases, and those with locally advanced disease. Chemotherapy has been considered to be the standard of care in cases with distant metastases, with no scope for local treatment. While the standard treatment of locally advanced disease is chemotherapy or chemoradiotherapy, their superiority or inferiority has not been clarified. The usefulness of combining chemotherapy with radiotherapy for local control is controversial [4, 5], but in some cases, additional radiotherapy leading to local control may lead to a long-term prognostic benefit [5–7]. Therefore, chemoradiotherapy is still considered as one of the effective treatment options for locally advanced unresectable pancreatic cancer in NCCN guideline [8].

In recent years, particle radiotherapy, including proton and carbon-ion radiotherapy, has become increasingly popular worldwide owing to its physical properties, which provide superior dosimetry and conformity to the target. This allows selective tumor irradiation, while minimizing doses to the gastrointestinal tract [9–11]. The efficacy of concurrent gemcitabine and particle radiotherapy for locally advanced pancreatic cancer has been reported in a phase I/II trial [12]. Shinoto et al. reported that the median survival time (MST) and 1-year overall survival (OS) rates in 72 patients treated with carbon-ion therapy for locally advanced pancreatic cancer were 19.6 months and 73%, respectively [13]. Terashima et al. reported that the 1-year local progression free survival, progression-free survival, and OS rates in 40 patients with locally advanced pancreatic cancer were 79.9%, 60.8% and 78.8%, respectively, after proton radiotherapy [12]. The escalated tumor doses delivered by radiotherapy offer better local control, and may positively impact prognosis [10, 12]. However, the pancreas is anatomically in close proximity to the stomach and duodenum. The doses delivered by proton beams for radical treatment are beyond the tolerance of the gastrointestinal tract. Therefore, the delivery of sufficient doses to the entire pancreatic tumor volume while maintaining conformity is difficult even with the sharp distribution of proton radiotherapy. In a study, 52.5% and 47.1% patients with cancers of the pancreatic head and body/tail, respectively, suffered radiation-induced ulcers in the stomach and duodenum after proton radiotherapy [14, 15]. To overcome this problem, we have since 2006 developed a new conceptual approach with space-making proton radiotherapy. It consists of spacer placement surgery and subsequent proton radiotherapy. In the previous report, a gel is used as a spacer in prostate cancer treatment, and its dosimetric advantage and biological compatibility have been discussed [16, 17]. Our team used a Gore-Tex sheet (W.L. Gore and Associates, Newark, DE) as a spacer, and inserted it between the tumor and the nearby gastrointestinal tract to reduce the dose to adjacent organs and increase the total tumor dose [18, 19].

The present study evaluated the technical aspects of surgical spacer placement and assessed the dosimetric changes of proton beams before and after surgical spacer placement in patients with locally advanced pancreatic body and tail cancer. In addition, this study investigated the safety, efficacy, and long-term outcomes of surgical spacer placement and subsequent proton radiotherapy in these advanced pancreatic cancers.

## Methods

### Patients

The medical records of 9 patients who underwent proton radiotherapy at the Hyogo Ion Beam Medical Center after surgical spacer placement at the Kobe University Hospital for locally advanced pancreatic body and tail cancers, between October 2007 and January 2018, were analyzed. In these patients, total doses were restricted by the close proximity of the tumor to the gastrointestinal tract, which necessitated the placement of surgical spacers prior to proton radiotherapy. The general condition of each patient before both surgical spacer placement and proton radiotherapy was evaluated using the Eastern Cooperative Oncologic Group-Performance Status scores; tumor stage was defined according to the Union for International Cancer Control tumor-node-metastasis classification, 8th edition [20]. All patients underwent contrast-enhanced computed tomography (CT) of the abdomen and chest, and positron emission tomography with 18F-fluorodeoxyglucose (FDG-PET) to rule out distant metastases and to define tumor stage. Blood samples were collected for the routine evaluation and assessment of tumor markers.

This study complied with the standards of the Declaration of Helsinki and the current ethical guidelines, and was approved by the institutional ethics board.

### Eligibility for treatment

The inclusion criteria for this study were as follows: (1) pancreatic cancer confirmed histologically or clinically by diagnostic imaging, such as ultrasonic endoscopy and/or CT and/or magnetic resonance imaging (MRI), (2) locally advanced unresectable pancreatic cancer without distant metastasis, confirmed by CT and/or MRI and/or FDG-PET, (3) an Eastern Cooperative Oncologic Group-Performance Status score of 0–2, and (4) cases where radical doses of proton radiotherapy could not be delivered owing to the proximity of the tumor and adjacent gastrointestinal tract. Patients with the following conditions were ineligible for proton radiotherapy: (1) obvious gastrointestinal invasion by the tumor, confirmed by gastrointestinal endoscopy and/or CT and/or MRI, (2) refractory ascites with minimal therapeutic efficacy, and (3) an Eastern Cooperative Oncologic Group-Performance Status score of 3–4. Patients who fulfilled the inclusion and exclusion criteria and provided written informed consent for surgical spacer placement and subsequent proton radiotherapy were enrolled.

### Treatment strategy

**Fig. 1:**
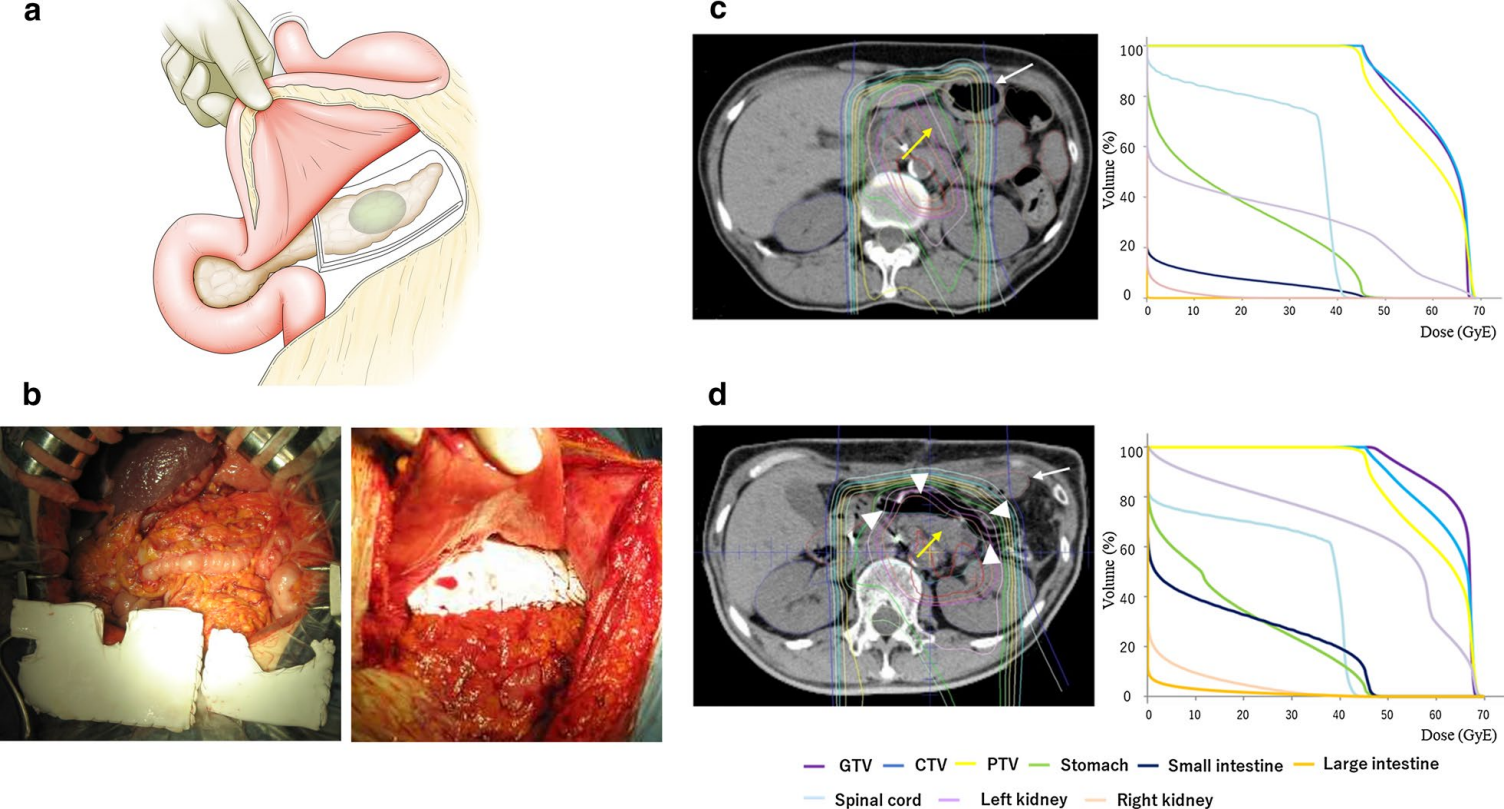
Images of representative patients who underwent surgical spacer placement. **a** Concept of surgical spacer placement. **b** Operative finding shows that surgical spacer (Gore-Tex sheet) was trimmed and interposed between the pancreas and gastrointestinal tract, and applied as a spacer after suturing tightly to the anterior surface of the pancreas and surrounding tissues. **c** Preoperative CT image and dose volume histogram reveal that the dose intensity for the gross tumor volume, and the clinical and planning target volume was restricted due to the close proximity of the pancreas (yellow arrow) to the stomach (white arrow). **d** Postoperative CT image and dose volume histogram reveal that a sufficient space between the pancreas (yellow arrow) and stomach (white arrow) was obtained by surgical spacer (arrowhead), and improved the dose intensity for the gross tumor volume, and the clinical and planning target volume while respecting the dose constraints of the gastrointestinal tract

The concept of surgical spacer placement is shown in Fig. 1a. The treatment strategy involves shielding of the dose of the gastrointestinal tract by placing a spacer during the first stage of the operation; this then enables the application of proton radiotherapy in the second stage.

### Surgical spacer placement

Surgical spacer placement was performed in cases in which proton radiotherapy could not deliver adequate curative doses to the pancreatic tumor as it was situated adjacent to the gastrointestinal tract. A Gore-Tex sheet was used as a surgical spacer to keep the gastrointestinal tract away from the tumor. It was placed between the anterior surface of pancreas and the deeper surface of the gastrointestinal tract. Since this procedure was only intended to allow the delivery of curative doses of proton radiotherapy to the tumor, no part of the tumor or gastrointestinal tract was resected or anastomosed during the spacer placement surgery. A foreign material, a Gore-Tex sheet, was inserted into the peritoneal cavity. After laparotomy, the presence of any liver metastases or peritoneal dissemination was excluded prior to surgical spacer placement. The subsequent steps were as follows. First, in cases without distant metastases, the omentum was incised from the mesenteric attachment of the transverse colon to open the reticular sac. Second, the splenic flexure of the colon was mobilized to reveal the space between the pancreas, spleen, and colon. Thirdly, four folded Gore-Tex sheets (20 cm × 15 cm × 2 mm) were interposed between the pancreas and gastrointestinal tract and applied as a spacer after suturing tightly to the anterior surface of the pancreas and surrounding tissues. This increased the distance between the gastrointestinal tract and the tumor (Fig. 1b). Fourth, physiological adhesions between the jejunum and the posterior segment of the colonic mesentery from the Treitz ligament were released in order to provide as much space as possible. Fifth, the trimmed omentum was placed and fixed in this space between the jejunum and the posterior segment of the colonic mesentery. An intraoperative abdominal cone beam CT examination was performed after surgical spacer placement to confirm whether the spacer was placed appropriately between the tumor and the gastrointestinal tract. The appearance in a typical case is presented in Fig. 1.

### Proton radiotherapy

Patients were treated with proton beams of 150–210 meV during the exhalation phase using a respiratory gating system; set-up was performed daily before irradiation using fiducial markers and bony landmarks. Treatment planning was performed on a CT-based three-dimensional treatment planning system (Xio-M; Mitsubishi Electric Corporation, Tokyo, Japan). The gross tumor volume (GTV) was defined as the primary tumor with the involved lymph nodes as determined on fused contrast-enhanced CT and FDG-PET images. The clinical target volume (CTV) comprised the GTV with a uniform 5-mm margin; the draining lymph nodes, para-aortic lymph nodes, and peripheral regions surrounding the celiac and superior mesenteric artery were irradiated to prophylactic doses. The planning target volume (PTV) was defined as the CTV with additional setup and respiratory gating margins of 5 mm and 1–5 mm, respectively. Total delivered doses were calculated according to the relative biological effectiveness (RBE). On the basis of biological experiments, the RBE value for the treatment beam was 1.1 [21]. Total doses of 70.2 Gy (RBE) in 26 fractions or 67.5 Gy (RBE) in 25 fractions were administered using the field-in-field technique. With this technique, we used three split doses—2 + 0.7, 1.8 + 0.9, and 1.6 + 1.1 Gy (RBE). For example, we delivered 1.8 Gy (RBE) to the whole PTV and 0.9 Gy (RBE) to the PTV excluding the gastrointestinal tract (the stomach, small bowel, and large bowel) in one fraction. Consequently, a maximum dose of 2.7 Gy (RBE) was administered as a single fraction (total, 67.5–70.2 Gy [RBE]) to the majority of the PTV while limiting the dose to the gastrointestinal tract to approximately 1.8 Gy (RBE) (total, 45 Gy [RBE]) [12]. The maximum dose to the highest irradiated 0.5 cc of an organ volume, namely, the stomach and duodenum, was limited to 48 Gy (RBE), and the maximum dose to the spinal cord was 45 Gy (RBE). For treatment delivery, we have used the passive beam formed by the bolus. During the irradiation period, CT was performed biweekly to confirm the positional relationship between the pancreas, spacer, and gastrointestinal tract, while treatment replanning was performed if necessary.

### Concurrent chemotherapy regimen

Concurrent chemotherapy was administered as gemcitabine monotherapy. Based on the dose and schedule of gemcitabine in previous studies [10, 22, 23], gemcitabine was administered at a dose of 800 mg/m^2^ via an intravenous infusion over 30 min on days 1, 8, and 15 for the initial 3 weeks of the 5-week proton radiotherapy schedule. Chemotherapy was administered provided the absolute granulocyte count was > 2000/mm^3^ and the platelet count was > 70,000/m^3^ on the scheduled day.

### Study design

For dosimetric comparison, pre- and post- spacer placement CT images were acquired for each patient. Target volumes and organ at risk were re-delineated on each series of the pre- and post-spacer placement images and treatment plans were created. To facilitate evaluation of dosimetric differences between pre- and post- spacer placement plans, the prescription dose was uniformly taken to be 67.5 Gy (RBE) in 25 fractions for the virtual dosimetric comparison study; this also applied to the patients who had received 70.2 Gy (RBE) in 26 fractions during treatment. To evaluate the efficacy and safety of surgical spacer placement, we analyzed the individual treatment plans using a dose-volume histogram (DVH) and compared them in terms of V95% (the volume irradiated by 95% of the treatment planning dose), V60 Gy (RBE) (the volume irradiated by more than 60 Gy [RBE]), D95% (the dose intensity covering 95% of the target volume), Dmean (average dose of the target volume), and Dmin (minimum dose of the target volume) of the GTV, CTV, and PTV; the D0.5 cc (maximum dose received by the highest irradiated 0.5 cc of the organ volume), V48Gy (RBE), V40Gy (RBE), and V30Gy (RBE) (percentage of the volume receiving at least 48, 40, and 30 Gy [RBE], respectively) of the stomach and intestinal tract were also assessed. Among these factors, the D0.5 cc and V48Gy (RBE) of the stomach and intestinal tract, and the V95% and V60Gy (RBE) of the GTV and CTV were indicative of adequate dose delivery to the target while respecting the dose constraints of the gastrointestinal tract after surgical spacer placement.

### Follow-up

After proton radiotherapy, all patients were examined every 3 months with repeated measurements of cancer tumor markers and contrast-enhanced CT and/or FDG-PET scans. As shown in previous reports [12, 24], local progression was defined as radiographic enlargement of the primary tumor, locoregional recurrence, or a tendency towards rising levels of tumor markers including carcinoembryonic antigen (CEA) and carbohydrate antigen 19–9 (CA19-9) for at least 3 months, without any distant metastases. The Common Terminology Criteria for Adverse Events (version 4.0) of the National Cancer Institute were used to define and grade the toxicities [25]. Complications were divided into 2 phases: (1) the early period: from surgical spacer placement to the completion of proton radiotherapy, and (2) the late period: after proton radiotherapy. To assess radiation-related gastrointestinal complications, all patients underwent gastrointestinal endoscopy from the esophagus to the horizontal part of the duodenum, before and every 3–6 months after proton radiotherapy.

### Statistical analysis

The MST and OS rates were calculated from the date of diagnosis and surgical spacer placement to the date of the event or last follow-up using Kaplan–Meier methods. Freedom from progression and local control rates were calculated from the date of surgical spacer placement to the date of the event or last follow-up using Kaplan–Meier methods. Differences in the survival rates between the groups were compared using the log-rank test. The unpaired Student’s t-test was used to compare the parameters of the dose-volume histograms. A value of *p* < 0.05 was considered statistically significant in all the analyses. All statistical analyses were performed using the JMP statistical software package, version 13 (SAS Institute, Japan).

## Results

### Patient characteristics and treatment course

The baseline patient and tumor characteristics are shown in Table 1. A total of 9 patients, comprising 6 women and 3 men were enrolled in this study. The median age of this group was 68 years (range: 44–86 years). All patients had an Eastern Cooperative Oncologic Group-Performance Status score of 0 or 1. The median tumor size was 34 mm (range: 21–60 mm). According to the tumor-node-metastasis classification, 4 (44.4%) and 5 (55.6%) patients had T4N0M0 and T4N1M0, respectively. The median serum levels of CEA and CA19-9 were 2.0 (range 0.8–5.1) ng/mL and 127 (range: 1–3267) U/mL, respectively. Table 2 shows treatment course and prognosis of all patients. As for the regimen of pre-treatment chemotherapy before spacer surgery, 3 (33.3%), 2 (22.2%), 1 (11.1%) and 3 (33.3%) patients had gemcitabine, Tegafur, Gimeracil, Oteracil Potassium (S-1), gemcitabine plus nab-paclitaxel (GNP) and none, respectively. Regarding the regimen of post-treatment chemotherapy after proton radiotherapy, 8 (88.8%) and 1 (11.1%) patients had gemcitabine and S-1, respectively.

**Table 1:** Patient and tumor characteristics

	No. of patients (*n* = 9)
Age, y, median (range)	68 (44–86)
Sex, *n* (%)	
Male	3 (33.3)
Female	6 (66.7)
ECOG^a^ performance status, *n* (%)	
0	3 (33.3)
1	6 (66.7)
Tumor size, mm, median (range)	34 (21–60)
Tumor invasion, *n* (%)	
Celiac artery	8 (88.9)
Common hepatic artery	9 (100)
Superior mesenteric artery	1 (11.1)
Splenic artery	9 (100)
Portal vein	6 (66.7)
Splenic vein	8 (88.9)
Anterior peripancreatic tissue	5 (55.6)
Posterior peripancreatic tissue	9 (100)
UICC TNM classification^b^, *n* (%)	
T4N0M0	4 (44.4)
T4N1M0	5 (55.6)
Postoperative serum laboratory data	
Albumin g/dl, median (range)	4.0 (3.7–4.4)
Pancreas-amylase IU/I, median (range)	47 (8–172)
Lipase IU/I, median (range)	33 (15–138)
CEA ng/ml, median (range)	2.0 (0.8–5.1)
CA19-9 U/ml, median (range)	127 (1–3267)

^a^Eastern Cooperative Oncology Group

^b^Union for International Cancer Control (UICC) tumor-node-metastasis (TNM) classification 8th edition

*CEA* carcinoembryonic antigen, *CA19-9* carbohydrate antigen 19–9

**Table 2:** Treatment course and prognosis of all patients

Age, Sex	Tumor size (mm)	Reason for UR-LA	Pre-treatment Regimen (course)	CA19-9 before surgery (U/ml)	Protocol GyE / Fr	Post-treatment regimen	Progression free survival (months)	Progression pattern	Status, survival time (months) after diagnosis / spacer surgery
67, M	50	CA, SMA	None	3267	Proton, 67.5 / 25	Gemcitabine	3	Peritoneal dissemination	Dead, 12 / 10
48, F	60	CA	Gemcitabine (2)	598	Proton, 67.5 / 25	Gemcitabine	9	Peritoneal dissemination	Dead, 16 /11
68, F	30	CA	S-1 (2)	35	Proton, 70.2 / 26	S-1	5	Peritoneal dissemination	Dead, 11 / 8
65, M	45	CA	None	1066	Proton, 67.5 / 25	Gemcitabine	NA	-	Dead, 10 / 9
55, M	34	CA	None	7	Proton, 70.2 / 26	Gemcitabine	18	Liver metastasis/Peritoneal Dissemination	Dead, 22 / 21
44, F	25	CA	S-1 (1)	102	Proton, 70.2 / 26	Gemcitabine	38	Local progression	Dead, 50 / 47
68, F	31	CA	Gemcitabine (1)	1	Proton, 67.5 / 25	Gemcitabine	16	Liver metastasis / Local progression	Dead, 31 / 30
68, F	21	CA	Gemcitabine (1)	127	Proton, 67.5 / 25	Gemcitabine	31	Lung metastasis	Dead, 52 / 50
65, F	46	CA, SMA	Gemcitabine plus nab-paclitaxel (6)	860	Proton, 67.5 / 25	Gemcitabine	NA	-	Alive, 14 / 7

*CA* celiac artery, *SMA* superior mesenteric artery, *S-1* Tegafur, Gimeracil, Oteracil Potassium, *NA* not available

### Changes in tumor volume and dose intensity before and after surgical spacer placement

Changes in tumor volume and dose intensity based on DVH before and after surgical spacer placement are shown in Table 3. The median time periods from the first medical examination to surgical spacer placement, and from surgical spacer placement to the irradiation of radiotherapy were 33 (range: 10–45) and 20 (range: 16–25) days, respectively. During these periods, the tumor volumes increased significantly (34.1 cc on first medical examination and 45.9 cc immediately before initiation of radiotherapy; *p* = 0.023).

**Table 3:** Changes in tumor volume and dose intensity based on dose volume histogram before and after surgical spacer placement

	Parameter	Preoperative Mean value	Postoperative Mean value	T-test
Tumor	Volume (ml)	34.1	45.9	0.023
GTV	V95% (%)	49.7	71.9	0.001
	V60(Gy [RBE]) (%)	66.4	84.7	< 0.001
	D95% (Gy [RBE])	49.7	53.7	< 0.001
	Dmean (Gy [RBE])	61.5	64.5	< 0.001
	Dmin (Gy [RBE])	46.2	48.1	0.019
CTV	V95% (%)	50.8	66.7	0.004
	V60(Gy [RBE]) (%)	64.1	78.4	0.002
	D95% (Gy [RBE])	48.0	50.1	0.005
	Dmean (Gy [RBE])	61.4	63.6	0.003
	Dmin (Gy [RBE])	44.6	45.2	0.030
PTV	V95% (%)	39.3	52.8	0.005
	V60(Gy [RBE]) (%)	51.1	65.2	0.002
	D95% (Gy [RBE])	45.3	45.9	0.021
	Dmean (Gy [RBE])	48.7	61.1	0.002
	Dmin (Gy [RBE])	37.9	37.1	0.451
Stomach	D0.5 cc (Gy [RBE])	46.6	45.8	0.190
	V48(Gy [RBE]) (cc)	0.211	0.16	0.144
	V40(Gy [RBE]) (cc)	22.3	12.2	0.018
	V30(Gy [RBE]) (cc)	40.0	27.5	0.038
Small intestine	D0.5 cc (Gy [RBE])	47.5	47.1	0.239
	V48(Gy [RBE]) (cc)	0.331	0.3	0.337
	V40(Gy [RBE]) (cc)	11.9	9.99	0.031
	V30(Gy [RBE]) (cc)	21.1	16.8	0.020
Large intestine	D0.5 cc (Gy [RBE])	20.7	14.0	0.228
	V48(Gy [RBE]) (cc)	0.068	0.0008	0.101
	V40(Gy [RBE]) (cc)	4.23	0.12	0.088
	V30(Gy [RBE]) (cc)	8.29	0.4	0.089

*GTV* gross tumor volume, *CTV* clinical target volume, *PTV* planning target volume, *RBE* relative biological effectiveness, *V95%* the volume that 95% of the treatment planning dose is irradiated, *V60* the percentage of volume irradiated more than 60 Gy (RBE), *D95%* a dose intensity covering 95% of the target volume, *D*_*mean*_, the average dose of the target volume, *D*_*min*_ the minimum dose of the target volume; D0.5 cc, maximum dose at which more than 0.5 cc of an organ volume is irradiated; V48, V40 and V30, the volume irradiated by more than 48, 40 and 30 Gy (RBE), respectively

In the GTV, CTV, and PTV, surgical spacer placement significantly improved V95% (*p* = 0.0012, *p* = 0.004, *p* = 0.005, respectively), V60Gy (RBE) (*p* < 0.001, *p* = 0.002, *p* = 0.002, respectively), D95% (*p* < 0.001, *p* = 0.005, *p* = 0.021, respectively) and Dmean (*p* < 0.001, *p* = 0.003, *p* = 0.002, respectively) while respecting the dose constraints of the gastrointestinal tract. In addition, the D0.5 cc and V48Gy (RBE) of the gastrointestinal tract tended to be reduced.

### Overall survival and local control

**Fig. 2:**
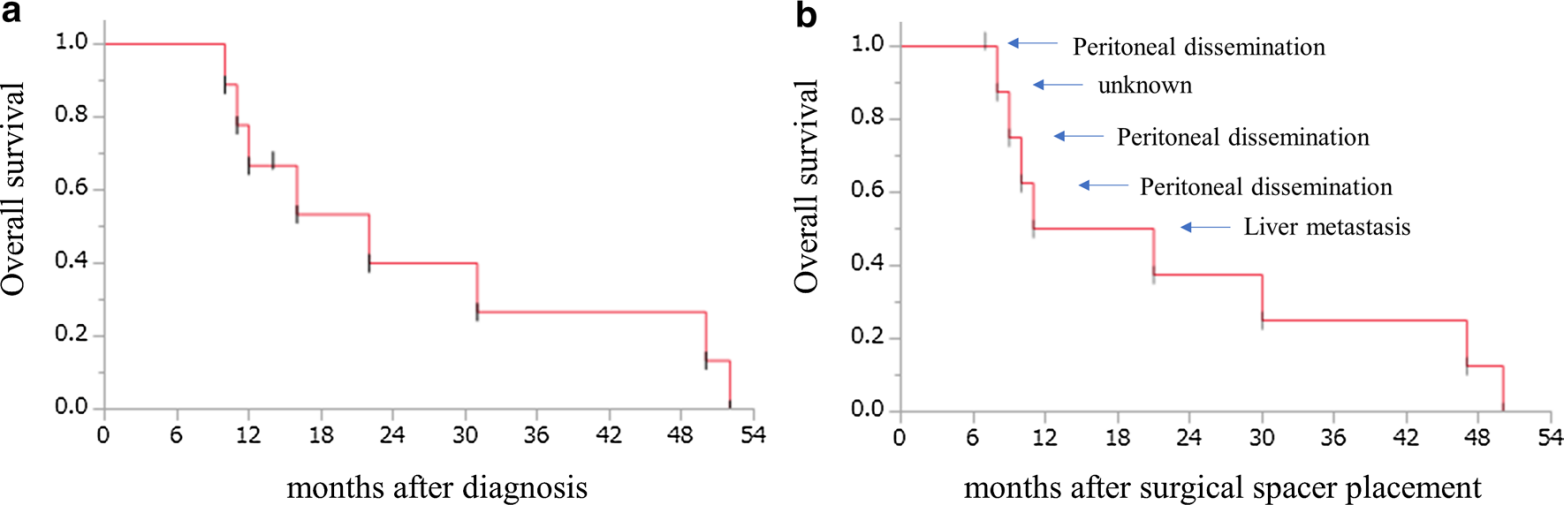
Overall survival rates of all 9 patients who underwent surgical spacer placement from the date of diagnosis (**a**) and surgical spacer placement (**b**) to the date of the event or last follow-up using Kaplan–Meier methods

**Fig. 3:**
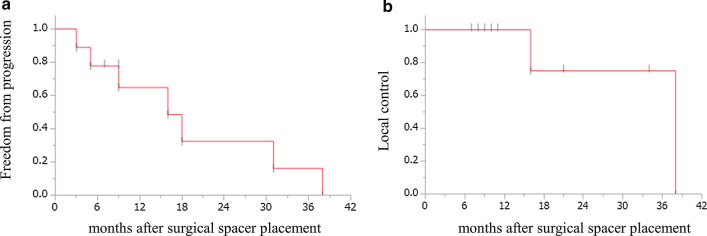
**a** Freedom form progression, and **b** local control rate of all 9 patients who underwent surgical spacer placement

The median period of follow-up from spacer surgery was 11 (range: 7–47) months; 8 of 9 patients died during this period. The OS rates are shown in Fig. 2. The MST after the date of diagnosis and surgical spacer placement were 22 (95% confidence interval [95% CI]: 10–50 months) and 16 months (95% confidence interval [95% CI]: 8–47 months), respectively. The freedom from progression and local control time were 16 (95% confidence interval [95% CI]: 3–38 months) and 38 months (95% confidence interval [95% CI]: 16–38 months), respectively (Fig. 3). The 1-year OS, freedom from progression, and local control rates after surgical spacer placement were 50.0% (95% CI: 20–80%), 64.8% (95% CI: 30–90%) and 100.0%, respectively. As shown in Table 2, 4 patients died within 1 year after spacer surgery. Among them, 3 patients died owing to peritoneal dissemination at 8, 10, and 11 months, respectively. The cause of death of the remaining 1 patient was unclear without tumor progression. Two patients who developed metastases to the liver and lung died at 21 and 50 months after spacer surgery, respectively. Conversely, local progression was observed in only 2 patients (22.2%); the time intervals to local progression were 16 and 38 months after spacer surgery, respectively (Fig. 3b). In patients with only local progression, OS time were 30 and 47 months after spacer surgery, respectively.

**Table 4:** Complications regarding surgical spacer placement and particle radiotherapy

	No. of patients (*n* = 9)
None, *n* (%)	2 (22.2)
Early period complications^a^	
Leukopenia	
Grade 1–2	0 (0)
Grade 3–4	2 (22.2)
Thrombocytopenia	
Grade 1–2	1 (11.1)
Grade 3–4	0 (0)
Dermatitis	
Grade 1–2	4 (44.4)
Grade 3–4	0 (0)
Abdominal pain	
Grade 1–2	3 (33.3)
Grade 3–4	0 (0)
Nausea	
Grade 1–2	3 (33.3)
Grade 3–4	0 (0)
Anorexia	
Grade 1–2	1 (11.1)
Grade 3–4	1 (11.1)
Late period complications^b^	
Gastrointestinal ulcer	
Grade 1–2	2 (22.2)
Grade 3–4	0 (0)
Gastric perforation	
Grade 1–2	0 (0)
Grade 3-4^c^	1 (11.1)
Abdominal infection by surgical spacer	
Grade 1–2	0 (0)
Grade 3–4	0 (0)

^a^Early period complications: adverse event during the period from operation of surgical spacer placement to end of particle radiotherapy

^b^Late period complications: adverse event during follow-up period after particle radiotherapy

^c^Gastric perforation occurred more than 4 months after particle radiotherapy

### Complications related to surgical spacer placement and proton radiotherapy

In patients with locally advanced pancreatic body and tail cancer, surgical spacer placement was technically feasible and was safely performed; subsequent proton radiotherapy was completed in all cases. The median duration of surgery and intraoperative blood loss was 191 min (median: 122–218 min) and 89 ml (range: 0–388 ml), respectively. Complications related to surgical spacer placement and proton radiotherapy in this group, according to the Common Terminology Criteria for Adverse Events version 4.0, are shown in Table 4.

A total of 7 (77.8%) of 9 patients had complications. In the early period, 2 (22.2%), 1 (11.1%), 4 (44.4%), 3 (33.3%), 3 (33.3%), 1 (11.1%), and 1 (11.1%) patients had grade 3–4 leukopenia, grade 1–2 thrombocytopenia, grade 1–2 dermatitis, grade 1–2 abdominal pain (owing to placement of the spacer), grade 1–2 nausea, grade 1–2 anorexia, and grade 3–4 anorexia, respectively. No patients developed gastrointestinal ulcers with hemorrhage.

In the late period, 2 (22.2%) patients developed gastrointestinal ulcers at 1 and 35 months after proton radiotherapy. These patients developed grade 1–2 ulcers in the horizontal part of the duodenum, which were healed with conservative management. However, 1 (11.1%) patient developed lower gastric perforation of an unknown etiology 4 months after proton radiotherapy. This patient required removal of the surgical spacer and total gastrectomy to manage the perforation. During the follow-up period, none of the patients with surgical spacers had infections due to long-term retention of a foreign material.

## Discussion

A multidisciplinary approach using surgical spacer placement and radiotherapy has been previously employed with conventional photon radiotherapy for other malignancies, such as rectal cancer [9, 26]. However, the inferior dose conformity of photon radiotherapy entails the use of unacceptable spacer volumes in order to realize this concept. Consequently, this approach did not become popular, and detailed reports, including dosimetric evaluation, have not been published to date. In contrast, owing to the sharp dose distribution of proton radiotherapy, a 10-mm distance between the tumor and adjacent gastrointestinal tract is sufficient for the safe delivery of therapeutic doses [27, 28]. We speculate that this combined approach may only be practically applicable with the clinical use of proton radiotherapy.

In this study, surgical spacer placement was technically feasible and was safely performed; subsequent proton radiotherapy was completed in all cases. Surgical spacer placement significantly improved the dose intensity for the GTV, CTV and PTV based on the DVH, while respecting the dose constraints of the gastrointestinal tract. Although the changes in the volume of CTV and PTV were not examined (because they are affected by various factors such as changes in the positional relationship of organs and the effects of spacer placement), the volume of CTV and PTV after spacer placement may have increased because of the absence of proximity to the gastrointestinal tract. There is a limitation to the interpretation of the result that the V95%, V60Gy, and Dmean of CTV and PTV have improved because they are affected by the volume changes. However, the improvement of all parameters of GTV, CTV, and PTV while respecting the dose constraints of the gastrointestinal tract may indicate the effectiveness of the spacer placement.

As for radiation-induced gastrointestinal toxicities, considering the beam settings which were planned to deliver maximal doses to be the GTV, CTV, and PTV while keeping the maximum dose to the highest irradiated 0.5 cc of organ volumes in the gastrointestinal tract limited to 48 Gy (RBE), the rate of radiation induced gastrointestinal ulcers is acceptable compared to previous reports [14, 15], and all of these cases were conservatively cured without bleeding. Additionally, good local control, which was reflected by the 1-year local control rate of 100% in this study, was achieved by surgical spacer placement for curative irradiation of proton radiotherapy. Surgical spacer placement enabled local treatment and control of locally advanced unresectable pancreatic body and tail cancer which was difficult for local control due to the proximity of gastrointestinal tract.

As for prognosis, OS, freedom from progression and local control time after spacer surgery in the present study was almost equal to that of previous studies, which included patients with locally advanced unresectable pancreatic cancer treated with curative dose of particle radiotherapy [12, 13]. It should be noted, that all patients in the present study were beyond inclusion criteria for proton radiotherapy due to the proximity of the gastrointestinal tract, this result may indicate that surgical spacer placement may contribute to deliver sufficient curative doses. Considering the good local control rates, reduced invasiveness, and wider treatable areas including the main tumor, neuro plexus, and perivascular tissue, some resectable pancreatic cancers with suspected vascular and nerve invasion may benefit from particle radiotherapy rather than surgery. Although this treatment strategy is currently indicated for locally advanced unresectable pancreatic cancer, expanding the indication to such cases should be explore in the future.

While good local control, there were also cases with poor prognosis due to potential micrometastasis. Actually, 3 of our 9 patients died due to peritoneal dissemination within 1 year of treatment despite good local control in this study. Cautious patient selection will be the next agenda for our treatment strategy. As in the NCCN guidelines, now that powerful regimen such as oxaliplatin, irinotecan, fluorouracil, and leucovorin (FOLFIRINOX) or GNP have emerged [7, 29, 30], induction chemotherapy (preferably 4–6 months) followed by chemoradiotherapy is recommended as one of the treatment options of locally advanced unresectable pancreatic cancer [8]. Under situation of distant control with powerful chemotherapy for locally advanced unresectable pancreatic cancer, treatment strategy of surgical spacer placement and subsequent proton radiotherapy can be an acceptable treatment option for locally advanced unresectable pancreatic body and tail cancer nearby gastrointestinal tract for further prolonging prognosis.

Surgical spacer placement and subsequent proton radiotherapy were associated with several problems. The first problem was tumor progression during the interval up to initiation of proton radiotherapy. For our treatment strategy, a median interval of 33 days elapsed between spacer placement and first irradiation. Shortening of this period is an issue; the period from spacer placement to first irradiation is approximately 10 days at present. The surgical limitations of spacer placement posed the second problem. In tumors invading the Treitz ligament, spacer placement is technically difficult due to insufficient space for the spacer material. The motion and range uncertainties during treatment was the third problem. Regarding the motion uncertainties associated with the Gore-Tex sheet, it is impossible to evaluate motion uncertainties during treatment without taking 4DCT. However, Gore-Tex sheets were placed between the pancreas and gastrointestinal tract and sutured tightly to the anterior surface of the pancreas and surrounding tissues. No case required a change in treatment protocol due to the positional disconnection of the spacer maybe due to the tight fixation and postoperative adhesions. Regarding the range uncertainties associated with the Gore-Tex sheet, it is difficult to experimentally determine the stopping depth inside the spacer of the proton beam in the patient’s body, thus, we could not evaluate it. Regarding the Gore-Tex sheet, we adopted conversion from the CT value to the stopping power ratio because the composition of the Gore-Tex sheet is almost the same as that of the human body (H, C, N, O, F, etc.) [31]. Therefore, we did not take any special consideration against the Gore-Tex sheet for treatment planning. However, since gastrointestinal mucosal damage did not occur, it is considered that there was no major problem with this calculation method. Adverse events associated with surgical spacer placement were the fourth problem. Adverse events included: (1) abdominal pain and discomfort consequent to surgical spacer placement, (2) infection risks due to the long-term retention of a foreign substance, and (3) risks of gastrointestinal tract perforation due to long-term contact with the surgical spacer. Although abdominal pain and discomfort were not apparent in all cases, analgetic drugs were required in some cases. In this study, no local infections occurred due to surgical spacer placement. However, infections have been recognized in cases of surgical spacer placement for other diseases. The most serious complication of this study was gastric perforation. Lower gastric perforation occurred at 4 months after proton radiotherapy, requiring removal of the surgical spacer and total gastrectomy. This may be a consequence of either inaccurate delivery of proton radiotherapy in the upper abdominal region due to respiratory fluctuations, or due to contact with the surgical spacer. In a study including patients who received particle radiotherapy without respiratory fluctuations for sacral chordoma, no gastrointestinal perforations were noted with surgical spacer placement. Absorbable surgical spacers are being developed for clinical applications; we have reported their usefulness in an animal model [32]. The material used in these spacers can dissolve within 3 months after placement. The clinical use of absorbable surgical spacers may reduce the incidence of adverse events such as perforation, infection and abdominal discomfort. Currently, clinical use of absorbable surgical spacers has started for retroperitoneal tumor close to the gastrointestinal tract. In the future, we are considering the clinical use of absorbable surgical spacers for pancreatic cancer nearby the gastrointestinal tract.

Although there are some problems to be solved, surgical spacer placement and subsequent proton radiotherapy were both technically feasible, and were safely completed in patients with locally advanced pancreatic body and tail cancer. In terms of DVH analysis, the efficacy of irradiation was significantly improved. In view of the better target coverage and improved local control offered by surgical spacer placement and subsequent proton radiotherapy, this may be a promising treatment option for locally advanced pancreatic body and tail cancer. In the future, careful patient selection and effective multimodal therapy may lead to improve prognosis in these patients.

## Conclusion

Comparing radiation plans, surgical spacer placement seems to improve dose distribution in locally advanced pancreatic body and tail cancer, which are in close proximity to the gastrointestinal tract. Further studies are necessary to assess the prognostic impact of surgical spacer placement and subsequent proton radiotherapy.

## Data Availability

The datasets used and analyzed during the current study are available from the corresponding author on reasonable request.
